# Absconding From Nonforensic Psychiatric Inpatient Settings: A Systematic Review of Risk Factors and Associated Patient Characteristics

**DOI:** 10.7759/cureus.110351

**Published:** 2026-06-06

**Authors:** Qaisar Farooq, Afreen Fatima, Ian French, Anwarul Haq

**Affiliations:** 1 Department of Psychiatry, St. Luke's General Hospital, Kilkenny, IRL

**Keywords:** absconding from psychiatric inpatient settings, department of psychiatry, mental health services, psychiatry and mental health, risk factors

## Abstract

Absconding from psychiatric inpatient units remains a significant challenge in mental health services and is associated with adverse outcomes, including interruption of treatment, relapse, self-harm, and disruption of continuity of care. This systematic review aimed to identify and synthesize the main risk factors associated with absconding among nonforensic psychiatric inpatients and to explore how these findings may inform clinical risk assessment and management. A systematic literature search was conducted using PubMed, EMBASE, and PsycINFO databases in accordance with the PRISMA guidelines, covering studies published from database inception to December 31, 2025. Studies involving adult patients in nonforensic psychiatric inpatient settings were included if they examined risk factors or patient characteristics associated with absconding. Eight studies met the inclusion criteria. Younger age was the most consistently reported risk factor across all studies. Other commonly identified factors included schizophrenia or other psychotic disorders, involuntary admission status, and the early phase of admission, particularly within the first two weeks. Male sex, environmental influences, poor insight, irritability, and previous absconding were also associated with increased risk. The findings suggest that absconding is a multifactorial phenomenon influenced by demographic, clinical, and environmental variables. Early identification of high-risk patients and implementation of targeted interventions may improve prevention strategies and patient safety in psychiatric inpatient settings.

## Introduction and background

Absconding, defined as leaving a psychiatric inpatient unit without permission, remains a significant clinical and organizational issue in mental health services and has been associated with interruption of treatment, relapse, self-harm, harm to others, and disruption of continuity of care [1,2]. In this review, nonforensic psychiatric inpatient settings refer to general adult psychiatric wards or acute mental health inpatient units that provide assessment, stabilization, and treatment for mental illness, excluding forensic, court-mandated, correctional, or high-security psychiatric services. It also places operational demands on mental health services because staff are required to initiate search procedures, inform relevant authorities, complete incident documentation, and undertake further risk management [1].

The reported prevalence of absconding varies widely across studies, reflecting differences in definitions, study populations, legal frameworks, ward design, and clinical settings [3,4]. Previous research has identified several factors associated with absconding, including younger age, male sex, psychotic disorders, particularly schizophrenia, and involuntary admission status [3,4]. Other factors, such as a history of previous absconding, substance use, and poor adherence to treatment, have also been suggested as potential predictors [3,5].

In addition to patient-related factors, environmental and situational influences appear to play an important role. Absconding events have been reported to occur more frequently during the early phase of admission and at times of reduced supervision, suggesting that ward organization, staffing patterns, and patient-staff interactions may contribute to risk [4]. Furthermore, qualitative research indicates that patients’ perceptions of restriction, dissatisfaction with care, and a desire for autonomy may influence the decision to abscond [6].

Despite these findings, the evidence base remains limited and heterogeneous. Variations in study design, definitions of absconding, and outcome measures make comparisons across studies difficult. Although factors such as younger age, psychotic disorders, and involuntary admission status have been repeatedly reported, the evidence remains heterogeneous because of differences in study design, definitions of absconding, clinical settings, and outcome measures. Therefore, an updated synthesis is needed to clarify consistent risk factors and identify areas where evidence remains limited.

Therefore, the aim of this systematic review is to synthesize the available evidence on absconding among nonforensic psychiatric inpatients, with a focus on identifying consistent risk factors and patterns. A clearer understanding of these factors may support improved risk assessment and inform strategies to reduce absconding in inpatient psychiatric settings. Therefore, the review question was: Among adults admitted to nonforensic psychiatric inpatient settings, what patient-related, clinical, legal, and environmental factors are associated with absconding?

## Review

Methods

This systematic review was conducted in accordance with the PRISMA guidelines [7]. A systematic search of PubMed, EMBASE, and PsycINFO was performed from database inception to December 31, 2025 to identify relevant studies examining absconding among psychiatric inpatients. The review protocol was not registered in PROSPERO or any other public registry.

Inclusion and Exclusion Criteria

The search results were screened by several independent reviewers using predefined inclusion and exclusion criteria. Studies were included if they met all of the following criteria: (1) the study was conducted in a nonforensic psychiatric inpatient setting; (2) the study used a prospective, retrospective, observational, comparative, or case-control design; (3) the study examined absconding from an inpatient psychiatric setting in relation to risk factors or associated patient characteristics; (4) the study reported extractable data on at least one demographic, clinical, legal, behavioral, or environmental factor associated with absconding; and (5) the article was published in English.

Studies were excluded if they were conducted in forensic psychiatric settings, general hospital settings without a psychiatric inpatient focus, nonpsychiatric inpatient settings or community-only settings or if they did not report relevant data on absconding risk factors or patient characteristics. Case reports, editorials, conference abstracts, review articles, and studies without sufficient outcome data were also excluded. Relevant data from eligible studies were extracted using a standardized template.

PRISMA Flow Diagram

**Figure 1:**
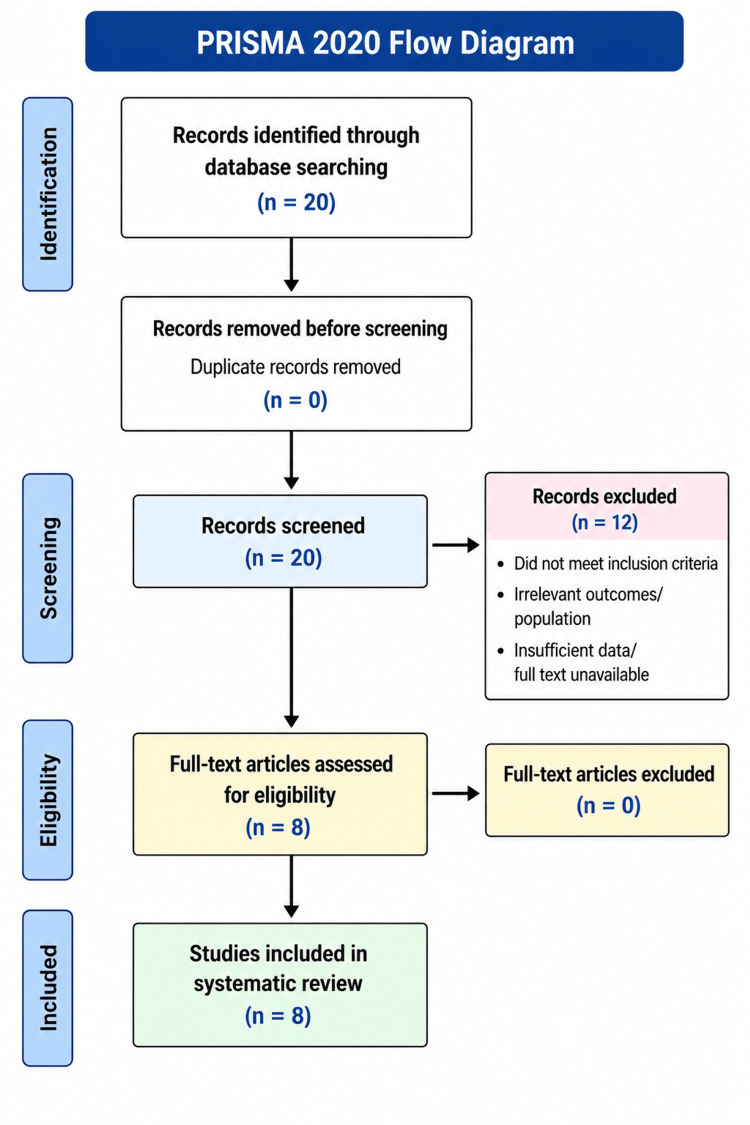
PRISMA flow diagram of study selection process

A PRISMA flow diagram was used to document the study selection process, including identification, screening, eligibility assessment, and final inclusion of studies (Figure 1). The study selection process was managed using Covidence systematic review software, which was used for duplicate removal, title and abstract screening, full-text review, and study selection.

The relatively small number of records identified reflects the focused scope of this review. The search was restricted to studies examining absconding in nonforensic psychiatric inpatient settings and reporting extractable data on risk factors or associated patient characteristics. Studies were excluded if they focused on forensic psychiatric populations, nonpsychiatric or community settings, did not examine absconding as the primary outcome, were review articles, editorials, conference abstracts, or did not provide extractable data relevant to risk factors. Following database searching and screening in Covidence, 20 records were identified for screening. Twelve records were excluded because they did not meet the predefined inclusion criteria, and eight studies fulfilled all eligibility criteria and were included in the final narrative synthesis. Therefore, the final number of included studies reflects the strict eligibility criteria and the specific nonforensic inpatient focus of the review rather than arbitrary exclusion.

Study Selection and Data Extraction

This study was conducted as a systematic review to identify and synthesize risk factors associated with absconding among psychiatric inpatients. Only studies published in English were included. In addition, reference lists of relevant articles were manually screened to identify further eligible studies, in accordance with established systematic review methodology [7].

Studies were eligible for inclusion if they examined absconding behavior in adult psychiatric inpatient populations within nonforensic settings and reported risk factors or associated patient characteristics using prospective, retrospective, or case-control designs. Studies focusing on forensic populations, nonpsychiatric settings, or those lacking relevant outcome data were excluded, consistent with previous research in this area [3,4].

All identified records were independently screened by three reviewers based on titles and abstracts, followed by full-text review according to predefined eligibility criteria, with disagreements resolved through discussion and consensus. Covidence systematic review software was used to manage duplicate removal, title and abstract screening, full-text review, and data extraction.

Characteristics of Included Studies

The characteristics of the included studies are summarized in Table 1. Previous literature has described patient-related, coercion-related, environmental, therapeutic, and risk-assessment factors associated with absconding and inpatient psychiatric care [8-15]. Owing to heterogeneity in study designs and outcome measures, a quantitative meta-analysis was not feasible; therefore, findings were synthesized using a narrative approach, with risk factors categorized into patient-related, clinical, and environmental domains, as described in previous studies [1,4]. As this review was based on previously published data, ethical approval was not required.

**Table 1:** Characteristics of included studies (N = 8) N refers to the total number of included studies. The sample size is presented as absconders/total participants. Sample denominators reflect the original study design. In case-control studies, the sample is presented as absconders/cases over the total case-control sample. In retrospective or prospective cohort/descriptive studies, the denominator represents the total inpatient population, admissions, or discharges reported in the original study.

Author (year)	Country	Design	Setting	Sample (absconders/total)
Bowers et al. (2000) [1]	UK	Case-control	12 wards (multicenter)	175/334
Mosel et al. (2010) [4]	Australia	Retrospective descriptive	Three wards	49/620
Meehan et al. (1999) [11]	Australia	Prospective observational	Single acute unit	51/390
Verma et al. (2020) [16]	India	Retrospective controlled	Psychiatric hospital	38/76
Khisty et al. (2008) [17]	India	Prospective	General hospital unit	33/231
Dickens et al. (2001) [18]	UK	Retrospective	Independent hospital	88/1466
Walsh et al. (1998) [19]	Ireland	Retrospective	Single hospital	95/1332
Gerace et al. (2015) [20]	Australia	Retrospective comparative	Single hospital	264/2184

Risk of Bias Assessment

Risk of bias assessment was conducted using the Newcastle-Ottawa Scale (NOS) for observational studies (Table 2) [21]. The NOS evaluates studies across three domains: selection of participants (up to four stars), comparability of groups (up to two stars), and ascertainment of outcomes or exposure (up to three stars), yielding a maximum score of nine stars. Studies scoring 7-9 were considered low risk, 4-6 moderate risk, and 0-3 high risk of bias. Each study was assessed across these three domains: selection of participants, comparability of study groups, and ascertainment of outcomes. Most studies demonstrated moderate methodological quality. Common limitations included retrospective study designs, small sample sizes, heterogeneity in definitions of absconding, and inconsistent reporting of clinical variables.

**Table 2:** Risk of bias assessment using the NOS Risk of bias was assessed using the NOS. Scores of 7-9 were considered low risk, 4-6 moderate risk, and 0-3 high risk. Full comparability scores were awarded only when studies clearly controlled for key confounders through matching, stratification, or multivariable adjustment. NOS, Newcastle-Ottawa Scale

Study	Study design	Selection (0-4★)	Comparability (0-2★)	Outcome/ascertainment (0-3★)	Total score (/9)	Overall risk of bias	Key limitations
Bowers et al. (2000) [1]	Case-control	★★★☆ (3/4)	★★ (2/2)	★★☆ (2/3)	7/9	Low	Large prospective case-control design; some residual confounding possible
Mosel et al. (2010) [4]	Retrospective descriptive	★★☆☆ (2/4)	☆☆ (0/2)	★★☆ (2/3)	4/9	Moderate	Descriptive design; small sample size; limited control for confounding
Meehan et al. (1999) [11]	Prospective observational	★★☆☆ (2/4)	★☆☆ (1/2)	★★☆ (2/3)	5/9	Moderate	Single-center study; no matched control group; limited generalizability
Verma et al. (2020) [16]	Retrospective controlled	★★☆☆ (2/4)	★☆☆ (1/2)	★★☆ (2/3)	5/9	Moderate	Single-center retrospective chart review; limited generalizability
Khisty et al. (2008) [17]	Prospective observational	★★☆☆ (2/4)	★☆☆ (1/2)	★★☆ (2/3)	5/9	Moderate	Short study period; context-specific findings from an Indian general hospital psychiatry unit
Dickens et al. (2001) [18]	Retrospective	★★☆☆ (2/4)	★☆☆ (1/2)	★★☆ (2/3)	5/9	Moderate	Retrospective design; limited statistical reporting for some outcomes
Walsh et al. (1998) [19]	Retrospective	★★☆☆ (2/4)	☆☆ (0/2)	★★☆ (2/3)	4/9	Moderate	Older dataset; limited reporting and limited control of confounders
Gerace et al. (2015) [20]	Retrospective comparative	★★☆☆ (2/4)	★☆☆ (1/2)	★★☆ (2/3)	5/9	Moderate	Retrospective design; dependent on routinely collected records

Results

A total of eight studies met the inclusion criteria and were included in the final analysis. The studies varied in design, including retrospective, prospective, comparative, and case-control approaches, and were conducted across different nonforensic psychiatric inpatient settings.

Across the included studies, several consistent risk factors for absconding were identified (Table 3). Younger age was the most consistently reported predictor, identified in all eight studies. Schizophrenia or other psychotic disorders, involuntary admission status, and the early phase of admission were each reported in six of the eight studies. Male sex was identified in five studies, although this association was less consistent.

**Table 3:** Predictors and key findings of absconding (N = 8) Findings summarized as reported in the original studies. LOS, length of stay; NS, not statistically significant

Author (year)	Key predictors	Statistical findings	Main findings
Bowers et al. (2000) [1]	Previous absconding, schizophrenia, male sex, younger age, nonadherence	OR 9.37; OR 3.11	Previous absconding was the strongest predictor
Mosel et al. (2010) [4]	Young age, involuntary admission, early-phase admission	NS (schizophrenia)	Institutional and staffing factors were relevant
Meehan et al. (1999) [11]	Young age, male sex, involuntary admission, schizophrenia, early-phase admission	Descriptive	Early admission and environmental factors; repeat absconding was common
Verma et al. (2020) [16]	Irritability, perceptual disturbance, poor insight, early-phase admission	p = 0.005; p = 0.04	Clinical symptoms were associated with absconding
Khisty et al. (2008) [17]	Schizophrenia, mood disorder, hallucinations, socioeconomic factors	p = 0.0202; p = 0.017	Clinical and socioeconomic factors were relevant
Dickens et al. (2001) [18]	Young age, involuntary admission, unmarried status	Not reported	No association with sex or LOS
Walsh et al. (1998) [19]	Young age, involuntary admission, schizophrenia, personality disorder	p < 0.001	Early admission and temporal patterns were associated
Gerace et al. (2015) [20]	Male sex, younger age, schizophrenia, longer LOS	OR 1.37; p < 0.001	Repeat absconding; early admission risk factors identified

Environmental and situational factors were identified in three studies, suggesting that ward structure, staffing patterns, and timing of events contribute to absconding behavior. Clinical symptoms such as poor insight and irritability were less frequently reported but demonstrated statistically significant associations where studied. Previous absconding, although identified in only one study, showed the strongest effect size, indicating its potential importance despite limited investigation.

Overall, the findings indicate that absconding is a multifactorial phenomenon influenced by a combination of demographic, clinical, and environmental factors, with no single predictor sufficient to explain all cases (Table 4). 

**Table 4:** Summary of risk factors for absconding (N = 8) Consistency reflects agreement across studies. No meta-analysis was performed because of heterogeneity.

Risk factor	Studies (n/N)	Consistency	Interpretation
Young age	8/8	High	Most consistent predictor across included studies
Schizophrenia/psychosis	6/8	High	Strong diagnostic association
Involuntary status	6/8	High	Frequently associated with absconding
Early phase of admission	6/8	High	Highest risk during the first days to the first one to two weeks of admission
Male sex	5/8	Moderate	Commonly reported, although not universal across studies
Environmental factors	3/8	Moderate	Ward structure, supervision, staffing patterns, and timing may contribute
Previous/repeat absconding	3/8	Moderate	Clinically relevant factor; strongest quantified effect reported by Bowers, with repeat absconding also described by Meehan et al. [11] and Gerace et al. [20]
Clinical symptoms	2/8	Low	Poor insight, irritability, and perceptual disturbance reported in limited studies
Unmarried/single status	1/8	Low	Reported as a significant demographic factor in Dickens et al. [18]
Nonadherence	1/8	Low	Identified in one study
Socioeconomic factors	1/8	Low	Context-specific finding
Personality disorder	1/8	Low	Limited evidence
Length of stay	1/8	Low	Longer admission was associated with absconding in one study; directionality remains unclear

Discussion

This systematic review identified a range of factors associated with absconding among psychiatric inpatients, supporting the view that absconding is a multifactorial phenomenon influenced by patient-related, clinical, and environmental variables. Consistent with previous literature, younger age emerged as the most robust and consistently reported predictor across all included studies. This finding aligns with earlier work suggesting that younger patients may demonstrate greater impulsivity, risk-taking behavior, and difficulty adapting to restrictive inpatient environments [1,8].

Psychotic disorders, particularly schizophrenia, were also strongly associated with absconding, as identified in the majority of included studies. This is consistent with prior research indicating that symptoms such as impaired reality testing, paranoia, and poor insight may contribute to a patient’s decision to leave the ward without permission [3]. Similarly, involuntary admission status was frequently associated with absconding, reflecting the complex relationship among perceived coercion, autonomy, and patient behavior in inpatient settings [4].

The early phase of admission, particularly within the first one to two weeks, was identified as a critical period of risk. This finding has been consistently reported in previous studies and may reflect both the severity of acute illness at presentation and the challenges associated with adapting to the inpatient environment [4,9]. Patients may experience distress, loss of autonomy, and dissatisfaction with care during this period, which may increase the likelihood of absconding [6,10].

Clinical features such as poor insight, irritability, and perceptual disturbances were also identified as relevant risk factors, although they were less consistently reported across studies. These findings are supported by previous research demonstrating that impaired insight and judgment are significant contributors to absconding behavior, as they affect patients’ understanding of their illness and the need for treatment [5]. Notably, previous or repeat absconding was reported in multiple included studies. Bowers et al. (2000) identified previous absconding as the strongest quantified predictor, while Meehan et al. (1999) and Gerace et al. (2015) also described repeat absconding as a clinically important finding, suggesting that past absconding behavior may be an important marker of future risk [1,11,20].

The findings of this review also support previous work showing that legal status and involuntary admission are important contextual factors in psychiatric inpatient care. However, forensic and nonforensic psychiatric settings differ substantially in legal mandate, security level, patient risk profile, length of stay, and discharge pathways. Therefore, risk factors identified in forensic settings may not be directly generalizable to nonforensic inpatient wards, where absconding may be more closely influenced by acute symptoms, perceived coercion, early admission distress, ward environment, and therapeutic engagement.

Environmental and situational factors were also highlighted in several studies. Ward structure, staffing levels, and periods of reduced supervision were associated with increased risk of absconding. These findings support the growing recognition that absconding is not solely determined by patient characteristics but is also influenced by the organizational context of care [4]. Previous studies have emphasized the importance of ward design, therapeutic engagement, and staff-patient relationships in mitigating absconding risk [8,13].

The findings of this review highlight the limitations of relying on a single “absconder profile” for risk prediction. While certain characteristics, such as younger age and psychotic disorders, are commonly observed, they lack sufficient specificity to reliably identify individuals at risk [1,14]. Instead, a comprehensive approach that considers multiple interacting factors is required. This is consistent with contemporary models of risk assessment in psychiatry, which emphasize dynamic and context-dependent factors rather than static predictors [15].

From a clinical perspective, these findings have important implications. Early identification of high-risk patients, particularly during the initial phase of admission, may allow for targeted interventions such as increased observation, enhanced engagement, and tailored care planning. Interventions aimed at improving patient experience, reducing perceived coercion, and strengthening therapeutic relationships may also help reduce the likelihood of absconding.

This review has several strengths. It focuses specifically on nonforensic psychiatric inpatient settings, which improves clinical relevance for general adult psychiatric wards. The review was conducted in accordance with the PRISMA guidelines and used multiple electronic databases, predefined eligibility criteria, and a structured data extraction process. It also synthesizes risk factors across patient-related, clinical, legal, and environmental domains, allowing a broader understanding of absconding as a multifactorial phenomenon. In addition, a formal risk of bias assessment was conducted using the NOS, strengthening the methodological appraisal of the included studies.

Limitations

This review has several important limitations that should be considered when interpreting the findings. First, there was considerable heterogeneity across the included studies in terms of study design, sample size, clinical settings, and definitions of absconding. Some studies defined absconding strictly as leaving without permission, while others included broader categories such as failure to return from leave, making direct comparison challenging. This variability limited the ability to synthesize findings quantitatively and precluded the use of meta-analysis.

Second, the majority of included studies were observational in nature, including retrospective and case-control designs. While these study designs are useful for identifying associations, they are inherently limited in establishing causal relationships. Confounding factors, such as illness severity, comorbid substance use, and variations in service provision, may not have been adequately controlled across studies, potentially influencing the reported associations. Third, sample sizes varied across studies, and in smaller single-center studies, the main methodological limitation was the limited use of multivariable adjustment and inconsistent inferential reporting rather than sample size alone. This restricts the ability to determine which factors are independently associated with absconding after accounting for potential confounders.

Fourth, retrospective documentation bias may have affected the assessment of clinical variables such as insight, irritability, and perceptual disturbance. In inpatient psychiatric records, behavioral markers and mental state abnormalities may be documented more thoroughly after an absconding event or behavioral escalation, making it difficult to determine whether these variables were present at baseline or emphasized retrospectively. This temporal reporting bias limits the validation of early-admission predictors. Fifth, publication bias is a potential concern, as studies reporting significant associations are more likely to be published than those with negative or inconclusive findings. In addition, absconding incidents may be underreported in clinical records or not systematically documented, particularly in retrospective studies, leading to potential reporting bias.

Sixth, restricting inclusion to English-language publications may have introduced language bias and may have excluded relevant studies published in other languages. Despite this limitation, the review provides a structured synthesis of the available evidence and highlights consistent patterns across studies, which may inform clinical practice and future research.

## Conclusions

This systematic review identified absconding from psychiatric inpatient settings as a multifactorial behavior driven by the interaction of patient-related, clinical, and environmental factors. Younger age was the most consistently reported risk factor, identified across all eight included studies. Schizophrenia or other psychotic disorders, involuntary admission status, and the early phase of admission, particularly the first one to two weeks, were each identified in six of the eight studies, representing the strongest and most replicated predictors. Male sex showed a moderate association, while previous/repeat absconding emerged as a clinically robust and important marker across multiple included studies, although it was underanalyzed using multivariable statistical methods. Clinical features such as poor insight, irritability, and perceptual disturbance, as well as environmental factors including ward structure and staffing patterns, were also relevant but were reported less consistently.

A key clinical implication is that no single “absconder profile” is sufficient for risk prediction. A dynamic, multifactorial approach to risk assessment, involving both patient characteristics and the care environment, is required. Early targeted interventions, particularly during the initial admission period, alongside improvements in therapeutic engagement and ward environment, may reduce absconding rates and enhance patient safety. Future research should prioritize prospective, multicenter studies using standardized definitions of absconding and consistent outcome measures. Studies should report timing of absconding, legal status, diagnosis, previous absconding history, substance use, insight, behavioral disturbance, ward environment, staffing patterns, and observation level in a uniform manner. Future studies should also use adjusted multivariable models to distinguish independent predictors from confounded associations. In addition, there is a need to develop and validate structured absconding risk assessment tools that incorporate both patient-related and environmental factors. Interventions targeting modifiable risks, such as early admission engagement, therapeutic relationships, observation practices, ward design, and staffing patterns, should be evaluated prospectively to determine their effectiveness and feasibility in routine inpatient psychiatric care.
